# Analysis of morbidity, mortality, and risk factors of tracheostomy-related complications in patients with oral and maxillofacial cancer

**DOI:** 10.1186/s40902-016-0078-9

**Published:** 2016-08-25

**Authors:** Seung Tae Lee, Min Gyun Kim, Jae Ho Jeon, Joo Hee Jeong, Seung Ki Min, Joo Yong Park, Sung Weon Choi

**Affiliations:** Oral Oncology Clinic, Research Institute and Hospital, National Cancer Center, 323 Ilsan-ro, Ilsandong-gu, Goyang-si, Gyeonggi-do 10408 Republic of Korea

**Keywords:** Oral cancer, Morbidity, Mortality, Tracheostomy complication, Risk factor

## Abstract

**Background:**

This study aimed to analyze and describe the morbidity and mortality associated with tracheostomy in patients with oral cancer and to identify the risk factors associated with tracheostomy complications.

**Methods:**

We performed a retrospective chart review of patients who underwent tracheostomy during a major oral cancer resection between March 2001 and January 2016 at the National Cancer Center, Korea. Overall, we included 51 patients who underwent tracheostomy after oral cancer surgery. We assessed the morbidity and mortality of tracheostomy and determined the risks associated with tracheostomy complications.

**Results:**

Twenty-two tracheostomy-related complications occurred in 51 patients. The morbidity and mortality rates were 35.2 % (*n* = 18) and 0 % (*n* = 0), respectively. Tracheostomy-related complications were tracheitis (*n* = 4), obstructed tracheostomy (*n* = 9), displaced tracheostomy (*n* = 5), air leakage (*n* = 1), stomal dehiscence (*n* = 1), and decannulation failure (*n* = 2). Most complications (19/22) occurred during the early postoperative period. Considering the risk factors for tracheostomy complications, the type of tube used was associated with the occurrence of tracheitis (*p* < 0.05). Additionally, body mass index and smoking status were associated with tube displacement (*p* < 0.05). However, no risk factors were significantly associated with obstructed tracheostomy.

**Conclusions:**

Patients with risk factors for tracheostomy complications should be carefully observed during the early postoperative period by well-trained medical staff.

## Background

Tracheostomy is a crucial surgical procedure to secure the perioperative airway in patients with oral cancer who undergo radical resection. However, tracheostomy has its own drawbacks as it is associated with serious complications that can sometimes be fatal. In a large review, the overall complication rate ranged from 6.7 to 48 %, with a mortality rate of 1.6 % [1]. However, recent reviews have reported lower rates of complications and mortality [2].

Alternative procedures for airway maintenance include endotracheal intubation or cricothyrotomy, and these less invasive procedures cause fewer complications [3, 4]. However, it is difficult to maintain cricothyrotomy and endotracheal intubation for long periods of time that is required for relief of postoperative swelling. Therefore, tracheostomy is considered an inevitable procedure, and it is commonly used after oral cancer surgery.

In the present study, we aimed to assess tracheostomy-related morbidity and mortality in patients with oral cancer who underwent radical operations and to evaluate the risk factors associated with tracheostomy complications.

## Methods

We performed a retrospective chart review of patients who underwent oral cancer ablative surgery at the Oral Oncology Clinic at the National Cancer Center in Korea from March 2001 to January 2016. Among all patients who underwent major oral cancer resection with free flap reconstruction, 51 patients who underwent tracheostomy for airway protection were included in this study. Tracheostomy was indicated for patients who required bilateral neck dissection, patients with tumors positioned at the back of the oral cavity or tongue and those who underwent anterior segmental mandibulectomy or resection of the floor of mouth. These patients were considered at risk of an immediate postoperative airway complication such as upper airway obstruction by postoperative tongue retrusion or sudden glottic edema. Tracheostomy was not usually performed in patients who underwent unilateral neck dissection.

The following variables were collected for each patient: age, sex, smoking history, alcohol use, weight, height, body mass index (BMI), tumor site, tumor pathology, neck height, pretracheal soft tissue thickness, type of neck dissection, and type of flap used. Complications were classified according to the time of their development (early or late), and they were graded as serious or minor. An early complication was defined as a complication that occurred within the first week of the tracheostomy, whereas a late complication was one that occurred 1 week after the procedure. Serious complications were potentially fatal, whereas minor complications were those in which patients required a hospital stay of less than 2 days. Only complications that could be directly attributed to tracheostomy were recorded.

The management protocol for all patients, regardless of the type of tube used, was very similar. Unless the patient had stridor or severe narrowing of the airway, nasotracheal or orotracheal intubation was performed. After oral cancer surgery, tracheostomy was performed by making a midline, 2.5-cm transverse incision at the lower neck. Then, the strap muscles were separated, and the thyroid isthmus was divided. Tracheostomy was performed by creating a window-type opening between the third and fourth tracheal rings. Additionally, the trachea was sutured to the skin (traction suture). After dilating the openings, a tracheostomy tube of the appropriate width and length was inserted by guidance of an inner obturator. The tracheostomy tube was inserted atraumatically by the lubricated end of the tube. When the cuff was promptly inflated, the end-tidal carbon dioxide and breath sounds were assessed. A single or double cannula tracheostomy was used depending on the attending surgeon’s preference. Patients were routinely managed in an intensive care unit (ICU) postoperatively.

The inspired air was humidified by a nebulizer, and the tube was regularly suctioned by an ICU staff experienced in post-tracheostomy care. All patients who underwent tracheostomy were managed by experienced nurses. Every patient received continuous humidified oxygen (5 L/min) by circuit through the tracheostomy tube, and suction was performed every 4 h and as needed. Tube patency was evaluated at 12-h intervals. The ICU respiratory therapists with additional experience and training in handling a tracheostomy tube block were available at all times for respiratory emergencies. Any noisy breathing, regardless of the type of tube used, was initially handled by the ward nurse by removing any mucus plug or blood clot by suction, if necessary. However, if the suction did not provide the clearance of the double cannula, the inner cannula was removed, cleaned thoroughly, and re-inserted. If diminished airflow through the tube continued despite these interventions or respiratory distress occurred, as indicated by a decrease in the oxygen saturation, tachypnea, or the use of accessory muscles for respiration, the attending physician and nurses with airway management experience were paged. If a tube block was confirmed, the tube was immediately removed. When a single cannula was used, the tube was changed every 2 days from the first postoperative day. The tracheostomy cuff was left deflated from the second day after the operation. After 1 week, the cuffed tube was replaced with a Koken tube. After using the Koken tube for 3 to 5 days, patients were assessed daily to determine whether it was safe to perform decannulation. If the posterior pharyngeal wall was clearly visible through the mouth with the tongue depressed, and the nasal fiber-optic assessment showed adequate oropharyngeal and laryngeal lumens with mobile vocal folds, it was considered safe to decannulate.

The frequency of tube changes differed for the single cannula and the double cannula tubes. Typically, the double cannula tube is replaced once per week. However, the inner cannula is replaced three times per day. There is no consensus or previous reports indicating the optimal frequency for cleaning the inner cannula. However, the cannula must be inspected on a regular basis, and this is usually performed at least three times per day, depending on the amount and consistency of the patient’s mucous secretions [5, 6]. Regardless of the type of tube used, the tube was completely removed after decannulation, and an occlusal dressing was applied to the tracheocutaneous fistula. Patients were monitored in the hospital for a minimum of 24 h following decannulation.

### Statistical analysis

The present study outcomes were the morbidity and mortality associated with tracheostomy complications and the risk factors associated with these complications. The chi-square test was used to determine the risk factors (categorical variables), and the Mann-Whitney test was used to analyze the continuous variables.

## Results

The present study included 51 patients (32 men and 19 women). The mean age was 53.87 years (range 10 to 78 years), and in most cases, tracheostomy was an elective procedure (98 %). Patient clinical characteristics, including cancer pathology, cancer site, cancer stage, and type of neck dissection, are presented in Table 1. Forty-four patients (86.2 %) were diagnosed as having squamous cell carcinoma, which is the most common pathology of the oral cavity. Of 51 patients, 32 underwent bilateral neck dissection. All patients required free flap reconstruction, and the following three types were used: fasciocutaneous flap (15 patients), musculocutaneous flap (30), and osteocutaneous flap (6).

**Table 1:** Clinical characteristics of patients that underwent tracheostomy (*n* = 51)

Pathology	Number of tracheostomies, *n* (%), *N* = 51
Squamous cell carcinoma	44 (86.27)
Osteosarcoma	3 (5.88)
Adenoid cystic carcinoma	2 (3.92)
Mucoepidermoid carcinoma	1 (1.96)
Ewing sarcoma	1 (1.96)
Sites of cancer	
Tongue	25 (49.01 %)
Alveolar ridge	10(19.60 %)
Floor of the mouth	7 (13.72 %)
Retromolar trigone	3 (5.88 %)
Buccal cheek	3 (5.88 %)
Others	3 (5.8 %)
Stage	
I	1 (1.96)
II	7 (13.72)
III	9 (17.64)
IV	34 (66.66)
Type of neck dissection	
Unilateral	19 (37.25)
Bilateral	32 (60.78)

*N* number

The morbidity and mortality rates were 35.2 % (18/51) and 0 %, respectively. Numbers and rates of early and late postoperative complications and minor and serious complications are shown in Table 2. Early postoperative complications occurred in 19 patients, and late postoperative complications occurred in three patients. Serious complications occurred in 12 patients, and minor complications occurred in eight patients. Twelve of 22 complications were serious (54.5 %): tube obstruction (*n* = 6), tracheitis (*n* = 3), displaced tracheostomy tubes (*n* = 1), and decannulation failure (*n* = 2). Additionally, only one patient in the double cannula group presented with a blockage that required tube removal, whereas eight patients in the single cannula group experienced a blockage that required tube removal. The rates of blockage with a double cannula and single cannula were 14.2 and 18.1 %, respectively.

**Table 2:** Tracheostomy-related complications

	Complications	Serious complications	Minor complications
Early postoperative period			
Tracheitis	4	3	1
Obstructed tracheostomy	9	6	3
Displaced tracheostomy	5	1	4
Air leakage	1	0	1
Late postoperative period			
Stomal dehiscence	1	0	1
Decannulation failure	2	2	0

Data in the table are the frequencies (number) of complication by severity

Tracheitis was only associated with the type of tube used (*p* < 0.05), whereas tube obstruction was associated with none of the variables. Tube displacement had a statistically significant relationship with patient BMI and smoking status (*p* < 0.05).

## Discussion

Tracheostomy is known for its complications and high mortality rate [7]. The reported complication rate in adults varies considerably [1, 8], and numerous alternative techniques to tracheostomy have been described [9–11]. Currently, the technical aspects of the tracheostomy procedure in an elective setting are considered safe, and this is supported by our study findings.

The absence of mortality in our patients can be attributed to the frequent tube changes and availability of emergency care by residents. The single cannula tube was changed on every other day, and the inner cannula of the double cannula tube was replaced three times per day.

Most cases of morbidity associated with procedure occurred during the early postoperative period. Of 12 serious complications, ten occurred during the early postoperative period, as shown in Table 2. There were nine cases of tube obstruction, which is an alarming complication for both the staff and patients. Eight of nine patients experienced tube obstruction with a single cannula, and the remainder (one patient) experienced tube obstruction with a double cannula. All of these events occurred within the first week of tracheostomy, and they were caused by a mucous plug, despite adequate humidification and suctioning. Tube obstruction can be prevented by proper suctioning, assessing the tube patency, and using a double cannula made of silicone. Silicone tracheostomy tubes do not stiffen after repeated use or after cleaning and disinfection. However, the silicone tubes should be inspected before each use, as cracks or tears may occasionally develop [12]. Patients and/or caregivers should be instructed on the proper technique for suctioning, including the frequency and indications for this intervention.

The use of premarked catheters is strongly recommended to ensure that the tube is inserted to a proper depth. Except under special circumstances, deep suctioning is not indicated, as the risk of epithelial damage increases. Suctioning should be performed depending on the clinical assessment of the patient. If there is no evidence of secretions, there is no need for extensive suctioning; however, suctioning in the morning and at bedtime and assessing the patency of the tube are always necessary.

Five tubes became displaced during the postoperative period. In one of those cases, the tube was inadvertently located outside the trachea. Generally, potential difficulties exist when surgical tracheostomy is performed in obese patients with short or thick necks. Morbidly obese patients with short and thick necks usually have abundant soft tissue between their trachea and skin. Unsuccessful blinded reintubation attempts may cause tube misplacement in the pretracheal areolar tissue, which can result in tracheal compression and respiratory arrest. However, pretracheal soft tissue thickness and tube displacement were not significantly associated in our study. Thus, the generally recognized relevance between the pretracheal soft tissue thickness and tube displacement conflicted with our findings. Further research with a larger sample sizes is necessary to clarify the association between pretracheal soft tissue thickness and tube displacement.

Two complications were classified as late complications. Two patients had decannulation failure because of an persistent upper airway obstruction. Additionally, stomal dehiscence occurred in one patient. As far as we know, stomal dehiscence has not been reported in any previous studies on tracheostomy complications, although it commonly occurs after laryngectomy [13]. Dehiscence may be caused by any form of tension on a patient’s short neck. This complication requires further investigation, particularly with regard to the timing of the tracheostomy.

Tracheitis, tube displacement, and tube obstruction were analyzed as outcome of risk factors. First, only the type of tube used was identified as an independent risk factor for tracheitis (Table 3). We considered that compared to a single cannula, a larger double cannula may cause more irritation to the posterior wall of the trachea. Regarding tube displacement, only the BMI and smoking status were independent risk factors (Table 4). BMI is related to obesity. In obese patients, there are many concerns regarding the technical difficulties of performing tracheostomy owing to the potential anatomic variations: palpation of the anterior neck landmarks [13], excessive submental and upper thoracic tissue obstruction of the surgical field, extension of the neck, thickness of tissues overlying the trachea, posteroanterior slant of the trachea owing to excess adipose tissue between the sternum and trachea, displacement of the airway structures inferiorly into the chest because of a short neck [14], and limitations for the placement of a standard tracheostomy tube curvature in relation to the anatomy in obese patients [15]. Additionally, there are medical concerns, such as a decreased pulmonary reserve, medical comorbidities, and other anesthetic risks [16]. Furthermore, history of smoking is an independent risk factor for chronic bronchitis, and frequent coughing is associated with the risk of tube displacement.

**Table 3:** Results of single-factor analysis of the risk factors for tracheitis

Variable	Tracheitis, present, *n* = 4 (78)	Tracheitis, absent, *n* = 47 (92.2)	*p* value
Patient-related characteristics			
Age, years, mean	53.0	54.8	0.624^a^
BMI, kg/m^2^, mean	25.3	22.2	0.064^a^
Sex			0.597
Male	3 (75.0)	29 (61.7)	
Female	1 (25.0)	18 (38.3)	
Smoker	3 (75.0)	27 (57.4)	0.493
Alcohol drinker	2 (50.0)	27 (57.4)	0.773
Neck height, cm	54.8	42.7	0.090^a^
Pretracheal soft tissue thickness, cm	9.8	11.6	0.266^a^
Treatment-related characteristics			0.597
Type of neck dissection			
Unilateral	3 (75.0)	29 (61.7)	
Bilateral	1 (25.0)	18 (38.3)	
Type of flap used			0.551
Fasciocutaneous	2 (50.0)	13 (27.7)	
Musculocutaneous	2 (50.0)	28 (59.6)	
Osteocutaneous	0	6 (12.8)	
Type of tube used			0.028
Single cannula	2 (50.0)	42 (89.4)	
Double cannula	2 (50.0)	5 (10.6)	

*BMI* body mass index

^a^Mann-Whitney test was used to analyze continuous variables. Data in the table are presented as numbers (percent), unless otherwise indicated

**Table 4:** Results of single-factor analysis of the risk factors for tube displacement

Variable	Displacement, present, *n* = 5 (9.8)	Displacement, absent, *n* = 46 (90.2)	*p* value
Patient-related characteristics			
Age, years, mean	60.6	54.0	0.378^a^
BMI, kg/m^2^, mean	25.5	22.1	0.038^a^
Sex			0.070
Male	5 (100.0)	27 (58.7)	
Female	0	19 (41.3)	
Smoker	5 (100.0)	25 (54.3)	0.049
Alcohol drinker	4 (80.0)	25 (54.3)	0.271
Neck height, cm	34.7	44.6	0.206^a^
Pretracheal soft tissue thickness, cm	13.4	11.2	0.298^a^
Treatment-related characteristics			
Type of neck dissection			0.070
Unilateral	5 (100.0)	27 (58.7)	
Bilateral	0	19 (41.3)	
Type of flap used			0.649
Fasciocutaneous	2 (40.0)	13 (28.3)	
Musculocutaneous	3 (60.0)	27 (58.7)	
Osteocutaneous	0	6 (13.0)	
Type of tube used			0.668
Single cannula	4 (80.0)	40 (87.0)	
Double cannula	1 (20.0)	6 (13.0)	

*BMI* body mass index

^a^Mann-Whitney test was used to analyze continuous variables. Data in the table are presented as numbers (percent), unless otherwise indicated

In the present study, there were no significant risk factors associated with tube obstruction (Table 5). Patients who undergo major oral cancer surgery involving a tracheostomy often have tracheostomy complications. Tracheostomy-related complications can be alarming because they can be life-threatening, prolong the recovery period, extend rehabilitation, and delay discharge from the hospital.

**Table 5:** Results of single-factor analysis of the risk factors for tube obstruction

Variable	Obstruction, present, *n* = 9 (17.6)	Obstruction, absent, *n* = 42 (82.4)	*p* value
Patient-related characteristics			
Age, years, mean	52.9	55.0	0.348^a^
BMI, kg/m^2^, mean	22.1	22.5	0.706^a^
Sex			0.789
Male	6 (66.7)	26 (61.9)	
Female	3 (33.3)	16 (38.1)	
Smoker	5 (59.5)	25 (55.6)	0.826
Alcohol drinker	4 (44.4)	25 (59.5)	0.407
Neck height (cm)	45.8	43.2	0.360^a^
Pretracheal soft tissue thickness (cm)	9.9	11.8	0.181^a^
Treatment-related characteristics			0.074
Type of neck dissection			
Unilateral	8 (88.9)	24 (57.1)	
Bilateral	1 (11.1)	18 (42.9)	
Type of flap used			0.384
Fasciocutaneous	1 (11.1)	14 (33.3)	
Musculocutaneous	7 (77.8)	23 (54.8)	
Osteocutaneous	1 (11.1)	5 (11.9)	
Type of tube used			0.802
Single cannula	8 (88.9)	36 (85.7)	
Double cannula	1 (11.1)	6 (14.3)	

*BMI* body mass index

^a^Mann-Whitney test was used to analyze continuous variables. Data in the table are presented as numbers (percent), unless otherwise indicated

## Conclusions

In patients who underwent surgery for oral cancer, tracheostomy complications occurred more commonly in the early phase (first week). During this early phase, special care is required to prevent potential catastrophic consequences of airway compromise. Therefore, intensive care by well-trained medical staff cannot be overemphasized during this period. Obese patients or those with short necks have increased risk of tracheostomy complications.
